# Bilirubin Prevents the TH^+^ Dopaminergic Neuron Loss in a Parkinson’s Disease Model by Acting on TNF-α

**DOI:** 10.3390/ijms232214276

**Published:** 2022-11-17

**Authors:** Sri Jayanti, Rita Moretti, Claudio Tiribelli, Silvia Gazzin

**Affiliations:** 1 The Liver-Brain Unit “Rita-Moretti”, Fondazione Italiana Fegato-Onlus, Bldg. Q, AREA Science Park, ss14, Km 163.5, Basovizza, 34149 Trieste, Italy; 2 Faculty of Medicine, University of Hasanuddin, Makassar 90245, Indonesia; 3 Molecular Biomedicine Ph.D. Program, Department of Life Sciences, University of Trieste, 34127 Trieste, Italy; 4 Neurology Clinic, Department of Medical, Surgical and Health Sciences, University of Trieste, 34139 Trieste, Italy

**Keywords:** tumor necrosis factor-alpha, neurodegenerative diseases, disease-modifying therapy, inflammation, redox, free bilirubin

## Abstract

Parkinson’s disease (PD), the fastest-growing movement disorder, is still challenged by the unavailability of disease-modifying therapy. Mildly elevated levels of unconjugated bilirubin (UCB, PubChem CID 5280352) have been shown to be protective against several extra-CNS diseases, and the effect is attributed to its well-known anti-oxidant and anti-inflammatory capability. We explored the neuroprotective effect of low concentrations of UCB (from 0.5 to 4 µM) in our PD model based on organotypic brain cultures of substantia nigra (OBCs-SN) challenged with a low dose of rotenone (Rot). UCB at 0.5 and 1 µM fully protects against the loss of TH^+^ (dopaminergic) neurons (DOPAn). The alteration in oxidative stress is involved in TH^+^ positive neuron demise induced by Rot, but is not the key player in UCB-conferred protection. On the contrary, inflammation, specifically tumor necrosis factor alpha (TNF-α), was found to be the key to UCB protection against DOPAn sufferance. Further work will be needed to introduce the use of UCB into clinical settings, but determining that TNF-α plays a key role in PD may be crucial in designing therapeutic options.

## 1. Introduction

Parkinson’s disease (PD) is the second-most common neurodegenerative disorder, with an annual incidence thought to be 11–19 per 100,000 [1,2]. Following the increase in the world’s aging population, the number of people affected is estimated to double by 2040, leading to the emergence of PD as a non-infectious pandemic [3]. To date, only symptomatic and temporarily effective therapies are available, making PD a high prevalence disease without an available cure [4]. This has escalated the urgency for finding new therapies that could slow down the disease progression, or even prevent its onset, even if its delayed diagnosis remains the major issue.

Bradykinesia, rest tremor, and rigidity are highlighted as the core of motor PD diagnosis [5]. These motor signs are due to the loss of TH^+^ positive dopaminergic neurons (DOPAn) in the substantia nigra pars compacta (SN), with a substantial proportion of TH^+^ DOPAn already lost (30–40%, Braak stage 2–3) [6] by the time the patient is diagnosed with PD [2].

Several mechanisms contributing to the disease have been demonstrated, both in humans and in experimental models. Specific genes (i.e., *SNCA*: alpha-synuclein, *LRKK2*: leucine-rich repeat kinase 2, *Parkin*, and *GBA*: glucocerebrosidase) have been determined to increase the susceptibility to the disease. Oxidative stress, mitochondrial dysfunction, neuroinflammation, and protein clearance disruption are among the most frequently reported molecular mechanisms [4,7]. Nevertheless, the key players in the etiopathogenesis of PD, and especially in the pre-diagnosis molecular steps leading to TH^+^ dopaminergic neurons number demise, remain unknown [4,7].

To study the chronic progressivity of PD, which requires decades in humans, a model representing the disease pathology and its corresponding mechanisms is needed. We developed a model with the ability to mimic the different stages of dopaminergic neuron sufferance, similar to human PD, within 96 h. We used an ex vivo (organotypic brain cultures of substantia nigra—OBCs-SN) progressing model of PD by challenging the slices with low doses of rotenone (Rot), a pesticide known to induce PD in humans and experimental models [8,9]. The major benefits of this PD ex vivo model consist of: (1) its closeness with the clinical scenario, mimicking in 24 h the progressive clinical stages up to the diagnosis, when the 30–40% of TH^+^ positive neurons are already lost (Braak stage 2–3), and then progressing to Braak stage 4–6, accompanied by the induction of microgliosis, apoptosis, etc. [6]. (2) Most importantly, the model gives us access to the early (pre-clinical stage) molecular events triggering TH^+^ positive neurons demise that can be the most effective targets of treatment. Among these events, we identified inflammation (interleukin 6—*Il6*, tumor necrosis factor alpha—*Tnfα*; cyclooxygenase 2—*Cox2*), redox imbalance (heme oxygenase 1—*Hmox1*, Sulfiredoxin 1—*Srxn1*), and growth factor imbalance (brain neurotrophic growth factor—*Bdnf*) as the early, potentially triggering mechanisms of TH^+^ positive neurons loss [8]. 

Unconjugated bilirubin (UCB, PubChem CID 5280352), an endogenous intermediate of hemoglobin metabolism conventionally known as a marker of hepatic dysfunction, has been recently proved to possess important roles in body homeostasis and against chronic pro-oxidant and pro-inflammatory diseases [10,11,12]. UCB has been proven to be a powerful antioxidant, even at nanomolar concentrations, where it can counteract 10,000 times higher levels of H_2_O_2_, and be even more powerful than α-tocopherol [13,14]. Moreover, it has been demonstrated to possess anti-inflammatory activities higher than those of dexamethasone [10,15,16]. As its roles have expanded as a ligand for various biologic cellular targets, most recently, UCB has been introduced as a hormone, strengthening the idea that the pigment has relevant physiological roles in the body [17]. The therapeutic potentials of UCB are observed in subjects with Gilbert syndrome (OMIM 143500), an inherited condition primarily due to a UGT1A1 (uridine glucuronosyltransferase 1A1) gene mutation which encodes the hepatic enzyme responsible for bilirubin clearance [10]. The mild hyperbilirubinemia in Gilbert syndrome offers health benefits linked with the reduced prevalence of non-CNS diseases, including cardiovascular disease, metabolic syndrome, non-alcoholic fatty liver disease, diabetes, and certain cancers [10,18,19]. In CNS diseases, UCB has been demonstrated to reduce neuronal damage in multiple sclerosis and glioma experimental models [20,21]. 

Currently, there is no experimental data available regarding the application of UCB in PD. To obtain a proof of concept on the therapeutic potential of low concentrations of UCB (Gilbert-like) in PD and to explore its molecular mechanisms of action, we assessed the action of bilirubin in our pre-characterized OBCs-SN model of PD [8]. 

## 2. Results

### 2.1. Low Levels of UCB Protect from the TH^+^ Positive Neurons Loss in the OBCs-SN Model of PD

**Figure 1:**
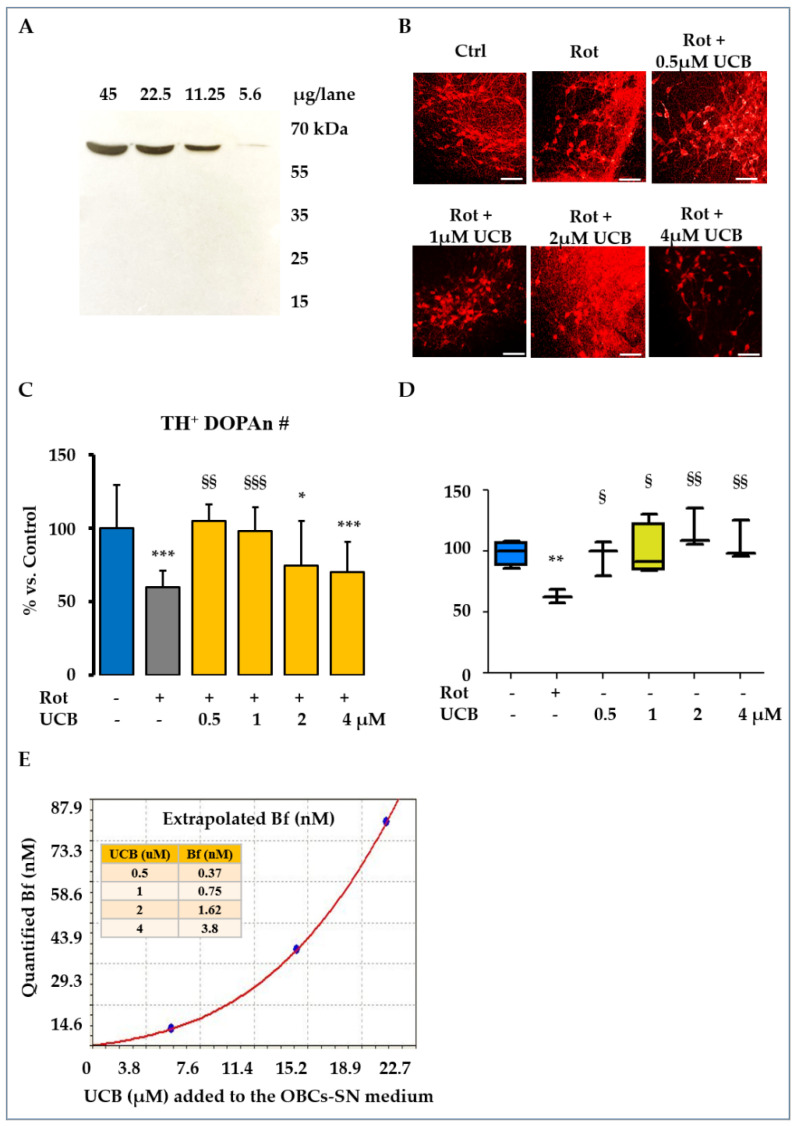
TH^+^ dopaminergic neuron (DOPAn) number in bilirubin-treated slices. To assess the effect of rotenone (Rot) challenging and unconjugated bilirubin (UCB) protection, the TH^+^ dopaminergic neuron (DOPAn) number (#) was counted in each treatment. (**A**) Western blot validation of the antibody in specifically detecting tyrosine hydroxylase (TH^+^) in our model. (**B**) Representative picture of tyrosine hydroxylase (TH^+^) DOPAn immunofluorescence (red signal); scale bar 100 μm. (**C**) Results of the counting of the TH^+^ DOPAn number under Rot challenging (grey bar, PD model) and Rot + UCB (from 0.5 μM to 4 μM) co-treatment (yellow bars) vs. control (Ctrl DMSO, blue bar, healthy slices). (**D**) Results of the counting of the TH^+^ DOPAn number in the OBCs-SN slices exposed to UCB alone. (**E**) Extrapolated free bilirubin (Bf) in the OBCs-SN media. In culture media containing albumin (carried by FBS), the moiety of bilirubin able to enter the cells is limited to the albumin-unbound UCB, called free bilirubin (Bf). Panel E shows the amount of Bf corresponding to each UCB amount we added to the OBCs-SN medium for performing the co-treatments in (**C**,**D**). Data are expressed as % vs. control. Data are expressed as mean ± S.D. for parametric data, and as the median quartile 1 and quartile 3 for non-parametric data. At least 4 independent repetitions were analyzed. Statistical significance: vs. Ctrl: * *p <* 0.05; ** *p <* 0.01; *** *p <* 0.001; vs. Rot: § *p* < 0.05; §§ *p* < 0.01; §§§ *p* < 0.001.

As depicted in Figure 1A, the antibody used allowed us to specifically detect the TH^+^ DOPAn in our slices. Figure 1B,C shows that the number of TH^+^ DOPAn under Rot challenge was reduced by 40% (Rot = grey bar; *p* < 0.001 vs. Ctrl = blue bar), in line with the clinical scenario of motor PD at diagnosis [22]. A dosage of 0.5 µM and 1 µM UCB (yellow bars, Figure 1C, corresponding to a Bf lower than 1 nM, Figure 1E), in co-treatment with Rot, fully reversed the DOPAn demise (*p* < 0.01 and *p* < 0.001 vs. Rot, respectively). A dosage of 2 and 4 µM UCB (both 2 µM and 4 µM of UCB corresponding to a Bf higher than 1.5 nM, Figure 1E) showed no statistical difference in respect to Rot, but was significantly different from the control (*p* < 0.05 and *p* < 0.001, respectively), indicative of a loss of protection. As shown by Figure 1D, the selected UCB concentrations alone (no co-treatment with Rot) were not toxic to the slices (no significant difference vs. control).

### 2.2. Screening for the Molecular Mechanisms of Protection

To understand the molecular mechanisms involved in the UCB protection at 0.5 µM and 1 µM, we have investigated the previously identified early potential triggers of DOPAn demise [8].

**Figure 2:**
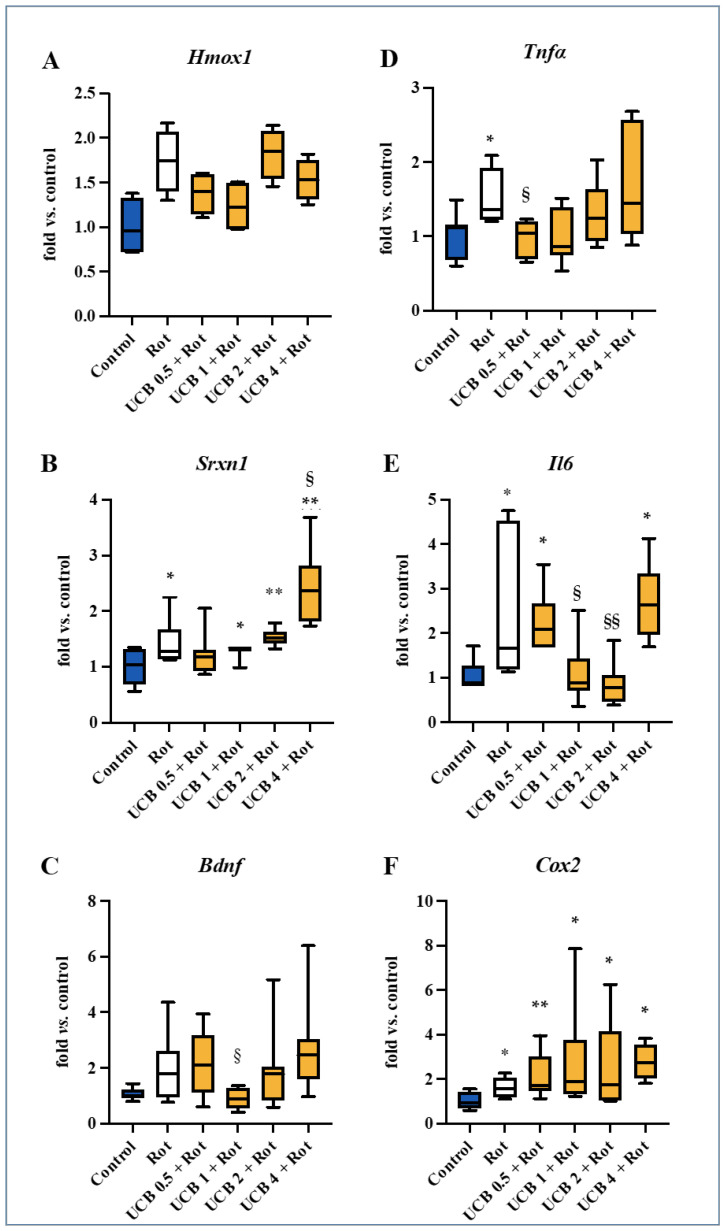
Evaluation of the changes in the early potential mechanisms of TH^+^ DOPAn loss. To evaluate the potential molecular mechanisms involved in UCB protection, we assessed the expression of the previously identified early molecular markers of DOPAn demise: (**A**) *Hmox1*: heme-oxygenase 1 and (**B**) *Srxn1*: sulfiredoxin 1 for redox stress; (**C**) *Bdnf:* brain-derived neurotrophic factor; (**D**) *Tnfα*: tumor necrosis factor alpha, (**E**) *Il6*: interleukin 6, and (**F**) *Cox2*: cyclooxygenase 2 for inflammation. Blue bar: DMSO control (healthy slices); grey bar: rotenone (Rot, PD); yellow bars: co-treatment of Rot + UCB from 0.5 μM to 4 μM. Data (mRNA expression) are expressed as fold vs. Ctrl, and as median, quartile 1, and quartile 3 of at least 3 independent repetitions. Statistical significance: vs. Ctrl: * *p* < 0.05, ** *p* < 0.01. vs. Rot § *p* < 0.05, §§ *p* < 0.01.

Because UCB is a well-known antioxidant [18,19,20,21], we first assessed the mRNA expression of *Hmox1* (heme oxygenase 1, a redox sensor) and *Srxn1* (sulfiredoxin 1, belonging to the battery of genes with an adaptive response to redox stress, and known to be induced by *Hmox1*) [23,24]. Rot treatment showed an upregulation trend for both redox markers vs. Ctrl (Figure 2A, Rot: grey bar; Ctrl: blue bar), which was statistically significant for *Srnx1* (*p* ˂ 0.05). Despite the lack of statistical significance, a trend in the reduction of the *Hmox1* level was observed with 0.5 μM and 1 μM UCB (Figure 2A, yellow bars), whereas 2 μM and 4 μM UCB were not able to downregulate the expression of the redox sensor.

None of the UCB treatments was able to normalize *Srnx1* expression, which was even increased at a higher UCB concentrations in respect to Rot challenging (4 μM UCB, *p* < 0.05 vs. Rot). 

Similarly, an increasing trend (vs. Ctrl) was present for brain-derived neurotrophic factor (*Bdnf*) after Rot exposure (Figure 2C). Dosages of 0.5 μM, 2 μM, and 4 μM UCB did not reversed this trend in respect to Rot challenging, while 1 μM was the only UCB concentration able to revert the *Bdnf* expression to the control level (*p* < 0.05).

Rot treatment also significantly increased the level of the inflammatory markers: tumor necrosis factor-α (*Tnfα*, Figure 2D), interleukin 6 (*Il6*, Figure 2E), and cyclooxygenase 2 (*Cox2*, Figure 2F; all *p* < 0.05). Treatment with 0.5 μM UCB reduced the *Tnfα* expression to the level of the control (Figure 2D, *p* < 0.05), while at 1–4 μM, the effect was lost. UCB induced a “U-shaped” normalization of the *Il6* up-regulation present (Figure 2E) in the Rot challenged slices, with 1 μM and 2 μM UCB fully reversing the *Il6* expression (*p* < 0.01 and *p* < 0.001 vs. Rot, respectively), with this effect not present at 0.5 μM and 4 μM UCB. No reduction effect was observed in *Cox2* levels (Figure 2F) with respect to Rot at all tested UCB concentrations.

By comparing the curve of DOPAn loss (Figure 1C) with the analysis of the molecular mechanisms triggering their demise (Figure 2A–F), both the trend of *Hmox1* and *Tnf-α* were in agreement with the protection conferred by UCB. Moreover, the *Srxn1* upregulation suggested a tentative response to an ongoing redox imbalance. Thus, we performed a Spearman’s rank correlation analysis between the TH^+^ DOPAn number and the mRNA expression of all selected markers. As depicted in Table 1, no significant results were found.

To investigate the involvement of these mechanisms in DOPAn loss in our model, we performed additional experiments.

### 2.3. Unravelling the Role of Redox Imbalance in the Model

**Figure 3:**
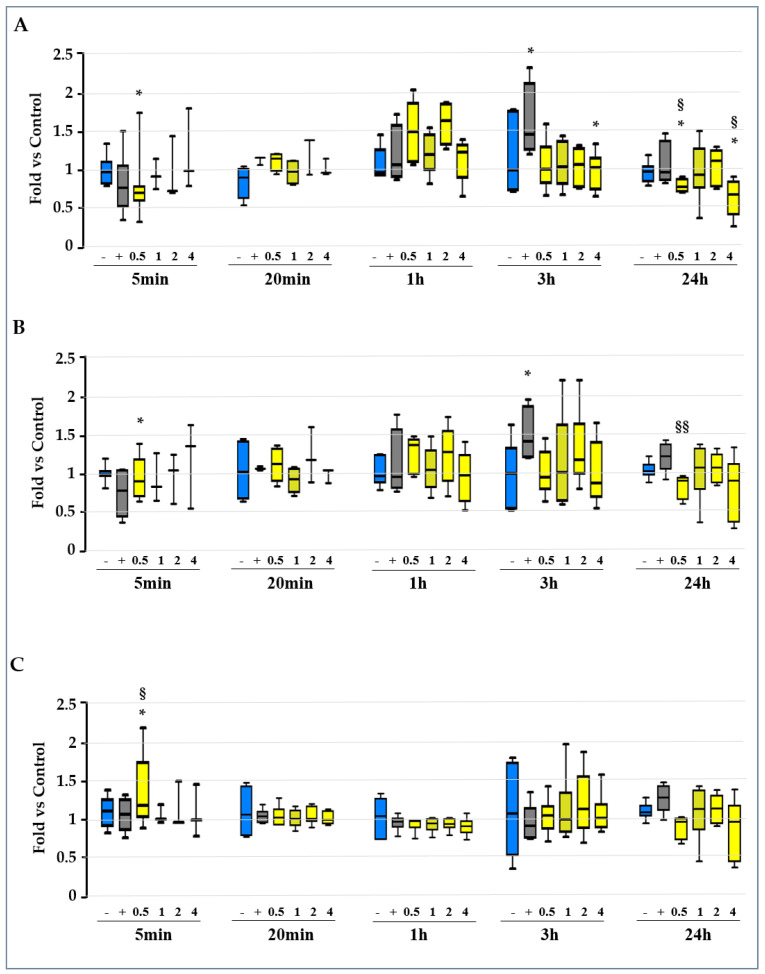
Time course of the reduced (GSH), oxidized (GSSG) glutathione and GSH/GSSG ratio in the OBCs-SN slices under Rot challenging and UCB protection. To deeply investigate the anti-oxidant action of UCB in protecting from DOPAn loss in our model, we calculated (**A**) the reduced/oxidized glutathione ratio (GSH/GSSG), from (**B**) the quantified reduced (GSH) and (**C**) oxidized glutathione content (GSSG). Due to the known rapidity of the induction of the redox imbalance, we performed a time course from 5 min after Rot/UCB addition, up to the final experimental timing (24 h). Blue bar: DMSO control (-: healthy slices); grey bar: rotenone (+: Rot, PD model); yellow bars: co-treatment of Rot + UCB 0.5 μM–4 μM. Data are expressed as fold vs. Ctrl, and as median, quartile 1, and quartile 3 of at least 3 independent repetitions. Statistical significance: vs. Ctrl: * *p* < 0.05. vs. Rot: § *p* < 0.05, §§ *p* < 0.01.

**Figure 4:**
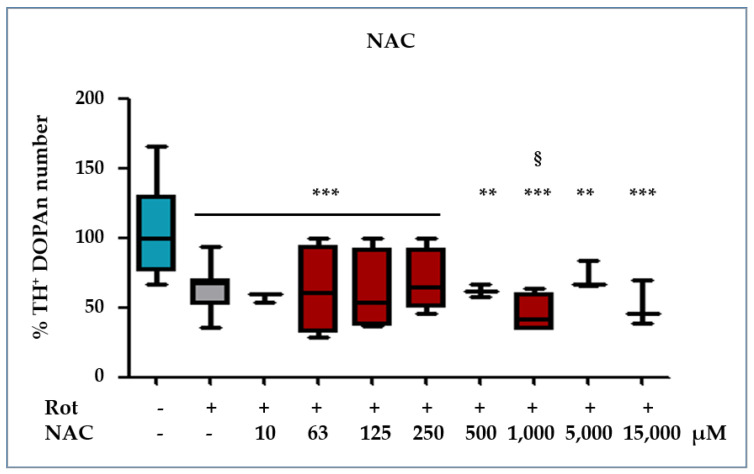
Effect of NAC treatment on TH^+^ DOPAn number. As additional proof of the minor involvement of oxidative stress in triggering TH^+^ DOPAn loss, slices were co-exposed to rotenone (Rot: grey bar, PD) and a wide range of N-acetyl cysteine (NAC) concentrations, encompassing all the potentially protective amounts found in the literature (orange bars). Blue bar: DMSO as a control (healthy slices). TH^+^ dopaminergic neuron (DOPAn) counting was used to assess the biological effect. Data are expressed as % of DOPAn number in exposed slices vs. the numbers of DOPAn in the control, and as median, quartile 1, and quartile 3 of at least 3 independent repetitions. Statistical significance: vs. Ctrl: ** *p* < 0.01; *** *p* < 0.001; vs. Rot: § *p* < 0.05.

To further investigate the involvement of the redox status in the early mechanisms triggering TH^+^ DOPAn reduction in our model, we added two sets of experiments. First, we investigated the glutathione contents in our cultures medium (Figure 3); then, to further clarify the role of oxidative imbalance on DOPAn demise, slices were co-exposed to Rot and a wide range of concentrations of N-acetyl cysteine (NAC, from 10 to 15,000 μM, Figure 4), a powerful antioxidant [25]. Because the induction in the redox stress is known to act quickly, we performed a time course from 5 min after Rot exposure up to the end of the experimental time (24 h).

#### 2.3.1. Evaluation of the GSH/GSSG Ratio, GSH, and GSSG Level along the Experimental Time

**Figure 5:**
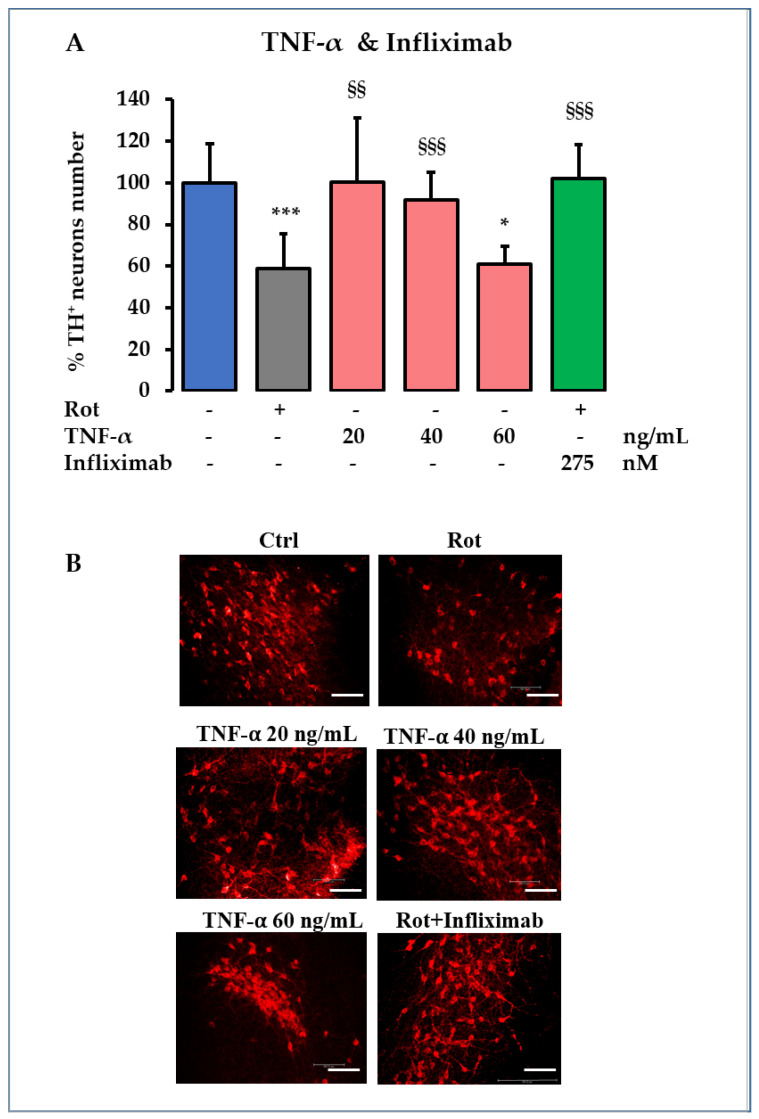
Demonstration of the determinant role of TNF-α in TH^+^ DOPAn demise. (**A**) To support the hypothesis that TNF-α is the determinant of TH^+^ DOPAn loss in our model, a range concentrations of TNF-α (pink bars) were added to the healthy slices (no rotenone). As counterproof, a TNF-α neutralizing antibody (Infliximab) was co-administered to Rot exposed cultures (green bar). Blue bars: control (DMSO); grey bar: rotenone (Rot, PD) challenging. TH^+^ DOPAn counting was used to assess the biological effect of the treatments. Data are expressed as % of the TH^+^ DOPAn number in exposed slices vs. the number of TH^+^ dopaminergic neurons in Ctrl, and as mean ± S.D. of at least 3 independent repetitions. Statistical significance: vs. Ctrl: * *p* < 0.05, *** *p* < 0.001; vs. Rot: §§ *p* < 0.01, §§§ *p* < 0.001. (**B**) Representative pictures of tyrosine hydroxylase (TH^+^) DOPAn immunofluorescence (red signal) in the different experimental conditions. Scale bar 100 μm.

As shown in Figure 3A, the GSH/GSSG ratio was reduced shortly after Rot challenge, despite not reaching statistical significance (5 min, gray bar). Similarly, a trend toward reduction was present in GSH, the protective form of glutathione (Figure 3B, 5 min, gray bar), but not in its oxidized form (GSSG, Figure 3C, gray bar). At this time, 0.5 μM UCB presented a significantly reduced GSH/GSSG ratio (Figure 3A—5 min, first yellow bar, *p* < 0.05 vs. Ctrl), due to a significant increase in the oxidized (GSSG) glutathione (Figure 3C—5 min, first yellow bar, *p* < 0.05 vs. both Ctrl and Rot). A 1 to 4 μM UCB co-treatment with Rot did not significantly change GSH/GSSG, GSH, or GSSG levels (Figure 3A–C—5 min, second and third yellow bars). The trend at 5 min did not agree with the DOPAn count. 

After 20 min and 1 h, no difference was found in the GSH/GSSG ratio (Figure 3A), nor in the GSH (Figure 3B) and GSSG (Figure 3C) content between the control, Rot, and UCB samples. 

At 3 h, the GSH/GSSG ratio increased in Rot challenged slices, due to an increase in the protective reduced form (GSH) (both ration and GSH *p* < 0.05 vs. Ctrl). Co-exposing the slices to Rot and UCB (from 0.5 to 4 μM) prevented the increase in both the GSH/GSSG ratio and the GSH content (4 μM, GSH/GSSG ratio, *p* < 0.05 vs. Rot, Figure 3A), indicating the absence of need for protection from Rot induced redox stress. 

Finally, at 24 h, UCB induced a significant reduction in the GSH/GSSG ratio at 0.5 and 4 μM (both *p* < 0.05 vs. both Ctrl and Rot), with a statistically relevant reduction in the GSH level vs. Rot at UCB 0.5 μM (*p* < 0.01 vs. Rot, Figure 3B).

#### 2.3.2. NAC Protection against the Rot-Induced Redox Imbalance

When slices were co-exposed to Rot and NAC, none of the used concentrations of NAC was able to increase the number of TH^+^ DOPAn (Figure 4), indicating that redox imbalance is not decisive in dopaminergic neuron sufferance.

Overall, these data pointed out that, despite its powerful antioxidant potential, the UCB ability to rescue TH^+^ DOPAn from Rot toxicity was not congruent with its antioxidant effect in our OBCs-SN model of PD. 

### 2.4. TNF-α Role in DOPAn Demise

Among all the inflammatory mediators modulated by Rot in our model, only *Tnfα* appeared to provide UCB-induced protection (see Figure 1C), suggesting a major role for TNF-α in the induction of TH^+^ DOPAn loss (under Rot) and protection (under Rot and UCB co-treatment) in our PD model. To demonstrate our hypothesis, we used two opposite approaches (Figure 5). First, we treated the OBCs-SN with TNF-α alone (Figure 5A, pink bars, in absence of Rot), and as the second approach, we co-treated OBCs-SN with Rot and Infliximab, a monoclonal antibody neutralizing TNF-α (Figure 5A, green bar) [26]. The TH^+^ DOPAn count (Figure 5A, blue: Ctrl, and grey: Rot) was used to assess the results of the two experimental settings.

Challenging OBCs-SN with TNF-α induced a concentration-dependent decrease in the TH^+^ DOPAn number, with 60 ng/mL TNF-α replicating the dopaminergic loss observed in the PD model (Figure 5A, TNF-α: pink bars, 40%, *p* < 0.05 vs. Ctrl: blue bar, compare with grey bar: Rot). Infliximab co-treatment with the pesticide fully protected DOPAn from Rot neurotoxicity (Figure 5, Infliximab: green bar, *p* < 0.001 vs. Rot: grey bar). 

The data confirmed that TNF-α is the key player in TH^+^ DOPAn loss in the model and that UCB confers protection by acting through *Tnfα* normalization.

## 3. Discussion

Delaying or stopping PD evolution is still an unmet medical need. Redox imbalance, and most recently, inflammation, are two discussed hits regarding TH^+^ DOPAn demise in PD patients and experimental models [4,7,8,27,28]. UCB possesses both antioxidant and anti-inflammatory properties, with demonstrated beneficial epidemiological effects in extra-CNS diseases [18,19]. What takes decades in the clinic may be achieved in a few days at the bench level. Our model is not acute, but is rather a progressing PD model able to mimic Braak stage 2–3 (30–40% of DOPAn demise) of human PD by using a low dose of Rotenone for 24 h treatment and later progressing to Braak stage 4–6. This allows us to gain access to the pre-diagnostic molecular events triggering TH^+^ DOPAn demise [8]. The measurement of the TH^+^ DOPAn number based on tyrosine hydroxylase positive (TH^+^) cell expression has been widely used in PD models and autopsies on humans with PD [8,29,30].

In this work, we demonstrated that low concentrations of UCB (0.5 μM and 1 μM, corresponding to a Bf lower than 1 nM) may provide up to a 40% protection from the TH^+^ DOPAn demise quantified in our ex vivo model of PD (Figure 1C). To decipher the mechanism of action of UCB, first, we focused our attention on its well-known anti-oxidant capability. The presence of redox imbalance in slices exposed to Rot is revealed by an altered GSH/GSSG ratio at 3 h (Figure 3A), well in agreement with similar models of PD) [31], and supported by the upregulation of *Srxn1*, belonging to the anti-oxidant response mechanism [24], and a trend toward increasing *Hmox1*, not only a redox sensor [32] and a transcription factor of the battery of anti-oxidant genes [24], but also the key enzyme in UCB production [10] (Figure 2A,B). An upregulation of *Hmox1* and an increased level of UCB in the serum have been reported in the early clinical stages of PD (Hoehn and Yahr Stage ≤ 3) [33,34], reversing with the progression of severity in the clinical setting. It was hypothesized that this double-sided trend represented an initial tentative reaction to the ongoing damage by increasing the in situ (CNS) production of bilirubin, failing later on with the progression of the disease [35]. As supported by the counting of DOPAn under the Rot and UCB co-treatments (Figure 1C), the addition of exogenous UCB to the cultures, possibly overcoming the need for *Hmox1* induction, conferred protection in our OBCs-SN. 

The longstanding *Srxn1* upregulation (up 24 h, Figure 2B) and the reduced GSH/GSSG ratio, both at 5 min (Figure 3A, 0.5 μM UCB) and at 24 h (0.5 and 4 μM UCB under UCB-Rot co-treatment) confirmed the presence of redox stress that bilirubin was not able to reverse, in disagreement with the counting of TH^+^ DOPAn (Figure 1). This data indicates that the primary protective mechanism of action of the pigment is not as an anti-oxidant, as well as that redox imbalance was not the determinant in the demise of the dopaminergic neurons.

The data was further supported by the NAC experiment (Figure 4), in which we were unable to normalize the TH^+^ DOPAn count in our model, despite the wide spectra of concentrations used. This point appears to be in disagreement with different models of PD, in which NAC has been reported to partially restore TH DOPAn loss [36]. The different result might be due to the different type of model (our model: OBCs-SN, other models: animals and cell lines) or, more plausibly, to the difference between acute and chronic conditions. Usually, high doses of Rot have been used to demonstrate the presence of oxidative stress and to develop an acute model of PD [29]. In contrast, by using a low dose of rotenone, we can induce the progression model of the disease [8], better reproducing the clinical scenario of a slow, chronic condition that requires decades to develop from a longstanding exposition to triggering stressors [27,28,37,38] and allowing for the discrimination between the early and late mechanisms triggering TH^+^ DOPAn demise [8]. These mechanisms can be the ideal target for treatments—still lacking in PD—aimed to modify the disease. Despite the fact that we detected the presence of oxidative stress in our PD model and that UCB possessed antioxidant activity, UCB protection was independent from its antioxidant activity, as indicated by the inability of NAC to prevent the rotenone-induced TH^+^ DOPAn reduction.

*Bdnf*, another potential contributor of TH^+^ DOPAn demise in experimental models [8,39,40], as well as in the clinical setting [41,42], was only normalized by 1 μM UCB (Figure 2C), disagreeing with the dopaminergic neuron protection observed in Figure 1C, showing protection never reached by BDNF administration in experimental models [43,44]. Similar results were observed with *Il6*, with a maximal normalization at 2 μM (Figure 2E), where UCB protection is lost (Figure 1C) and *Cox2*—never restored by UCB treatments (Figure 2F)—with long-term NSAIDs administration was not associated with a reduced risk of PD in chronic inflammatory diseases [45,46]. The experimental data from our OBCs-SN agree with different experimental and clinical models of PD, in which the alteration of all these markers has been noted [8,47,48,49,50,51]. Correlational studies on TNF-α levels and PD frequency in NSAIDs users have never been performed. The correlation between IL6 genetic polymorphisms increasing cytokine production and the risk of PD is unclear, keeping the question open regarding the relevance of this cytokine in the onset of PD [52,53].

TNF-α as a key contributor to PD onset and TH^+^ DOPAn demise is gaining more and more attention [28,38,54,55], but solid evidence of its relevance is still missing. Indeed, up until now, no effective drugs (in this work we used bilirubin as a drug [56]) have been found to reverse its expression and restore the TH^+^ DOPAn number. TNF-α modulation has been reported in patients [37,57,58], and is associated with a rapid motor decline [57]. These data have been replicated in models [8,47], with increased apoptosis [59] and TH^+^ DOPAn loss [60]. Experimental data obtained using an engineered TNF variant able to sequester the native cytokine from its receptor resulted in a partial (50%) rescue from TH^+^ DOPAn demise [38], possibly due to a partial efficacy in blocking the native molecule. Similarly, a small reduction in PD incidence has been reported in patients with inflammatory bowel disease exposed to Infliximab [61], possibly because of the drug’s limitation in crossing the blood–brain barrier [62].

In this study, we have documented that TNF-α is the determinant of both DOPAn demise and UCB protection in the model. While the coinciding of TH^+^ DOPAn number (Figure 1C) and *Tnfα* expression under Rot challenging and UCB treatment (Figure 2D) was speculative, the addition of purified TNF-α satisfactorily reproducing the neuronal loss and the full protection obtained by infliximab point to its key role in this process (Figure 5). Microglia, the immune resident cells of the CNS, are known to produce *Tnfα* enhancing TH^+^ DOPAn demise in PD. In our model, *Tnfα* upregulation preceded microglia activation, suggesting that other cells might be responsible for its production. In vitro models of PD have reported that DOPAn-like cell lines may produce cytokine, as well as astrocytes, known to release pro-inflammatory mediators. The identification of the cell type responsible for the early activation and release of Tnf*α* might be of therapeutic relevance. Studies using complex models (OBC-SN, in vivo) may help to answering these questions.

Clinical studies evaluating the incidence of PD in the Gilbert population are so far not available, but are required in light of our data. Notably and differently from the limited bioavailability of infliximab in the CNS, free bilirubin (Bf), the UCB moiety not bound to serum albumin, may quickly diffuse across the blood–brain barrier and the cells [63]. Nevertheless, converting the use of UCB as a drug in the clinic is not easy. Despite the fact that the systemic level of UCB may be modulated [64], its specific delivery to the substantia nigra of PD patients is a challenge. Nano-vehicles loaded with UCB may represent a feasible method [65,66]. Further experimental work is needed to translate the use of UCB findings into clinical settings. Nevertheless, our findings are useful for searching for new (prophylactic) therapeutic approaches able to specifically target TNF-α to prevent PD and potentially other neurodegenerative diseases sharing TNF-α as a contributor to neurodegenerative progression.

## 4. Materials and Methods

### 4.1. Organotypic Brain Culture Preparation

OBCs-SN were prepared as previously described by Dal ben et al. [8]. Wistar Han^TM^ rats, at 5 days after birth (P5), were obtained from the animal facility of the University of Trieste. The whole litter was used (the mean number of pups in the litter is 8). Animals were housed in a temperature-controlled environment (22 ± 2 °C), on a 12 h light/dark schedule, with ad-libitum access to food and water. Animal experiments were performed according to the Italian Law (decree 87-848) and European Community directive (86-606-ECC). The project was approved by the local ethics committee (Organismo unico Per il Benessere Animale—OPBA, Università degli Studi di Trieste) and by the Italian Ministry (code: 04086.N.VBU and 1FF80.N.PZB).

Shortly after sacrificing by decapitation, the rat brains were removed and maintained in the dissection medium (ice-cold Gey’s Balanced Salt Solution—Sigma-Aldrich St. Louis, MO, USA, plus D-Glucose 10 mg/mL) until use. The ventral tegmental area containing the SN was dissected and cut transversely into slices of 300 μm thickness by a McTwain Chopper (Gomshall Surrey, UK). Healthy slices were selected for the integrity of the structure under stereomicroscope visualization. The slices were seeded on sterile, semi-porous Millicell-CM inserts (PICM03050, Millipore, Darmstadt, Germany) and maintained in the culture medium containing 65% Basal Medium Eagle (Life Technologies Corporation, Grand Island, NY, USA), 10% Fetal Bovine Serum (FBS, Euroclone, Milan, Italy; containing albumin), 25% Hank’s Balanced Salt Solution (Sigma-Aldrich, St. Louis, MO, USA), 1% L-Glutamine (Euroclone, Milan, Italy), 2% Penicillin/Streptomycin (Life Technologies, Carlsbad, CA, USA), and 10 mg/mL D-Glucose (Sigma-Aldrich, St. Louis, MO, USA) until use.

### 4.2. Treatments

OBCs-SN were challenged with specific compounds 10 days after slicing, as previously described [8].

- A PD model of rotenone (Rot, R8875, Sigma-Aldrich, St. Louis, MO, USA), a pesticide known to trigger PD in humans and animal models [8,67], was used in this study. Rot was dissolved in DMSO (Sigma-Aldrich, St. Louis, MO, USA) and was used in a final concentration of 10 nM in the culture medium [8]. The final concentration has been experimentally identified for recreating a slow progressive demise of the TH^+^ DOPAn, mimicking the clinical scenario corresponding to the diagnosis in patients (40% TH^+^ DOPAn loss), after 24 h of challenge. At that time, the early mechanisms acting on DOPAn demise were maximally modulated by Rot [8]. For this reason, we chose 24 h as the time period to study their role and the potential protection conferred by bilirubin in this work.
- For the evaluation of UCB protection, UCB (B4126, Sigma-Aldrich, St. Louis, MO, USA. PubChem CID 5280352) was purified as previously described [68] and dissolved in DMSO to reach the desired concentrations. As preliminary data for this work, we carefully selected the concentrations of the potential protection conferred by UCB. Doses of 10 μM and 20 μM UCB were toxic to heathy slices, inducing TH^+^ DOPAn loss (60% and 70%, respectively); thus, they were excluded. Doses of 2 μM and 4 μM concentrations were not so high as to be toxic (adding toxicity to Rot challenged slices), but not so low as to be protective toward Rot-induced damage. The highest concentration of 4 μM UCB in the culture medium was determined using the spectrophotometer (absorbance of 0.190, λ = 440 nm). The final concentrations of 2 μM, 1 μM, and 0.5 μM UCB were reached by dilution; then, Rot was added to UCB-containing medium to perform co-treatments. The control medium was prepared by dissolving an equal amount of DMSO, the vehicle of both UCB and Rot (control: ctrl). Because FBS contains albumin, a binder of UCB, we quantified the amount of UCB not bound to albumin (namely, free bilirubin—Bf; the moiety of UCB entering the tissue) in the OBCs-SN medium, as described by Roca et al. [69]. All the selected UCB concentrations corresponded to a Bf < 4 nM, considered to be physiological concentrations, or Gilbert-like conditions [18,70].
- For the evaluation of redox imbalance to TH^+^ DOPAn loss, N-acetyl-cysteine (NAC, Sigma-Aldrich, St. Louis, MO, USA) treatment was used. We applied a broad range of NAC concentrations, based on the methods in the literature [71,72]. NAC was dissolved in medical water to a 125 mM concentration, then diluted it to the final concentrations ranging from 10 μM to 15,000 μM in the OBCs-SN medium, and Rot was added to perform co-treatments.
- For DOPAn loss by TNF-α challenging, TNF-α (Peprotech, London, UK) was diluted directly in the OBC-SN medium, starting with the TNF-α concentration suggested by the literature [38], and was increased at three concentrations (20 ng/mL, 40 ng/mL, and 60 ng/mL) until replication of the TH^+^ DOPAn loss observed in the Rot treatment was reached.
- For the infliximab protection in PD model, infliximab (Flixabi^®^, Biogen, Hillerod, Denmark), a TNF-α neutralizing antibody, was diluted in the OBC-SN medium at the final concentration of 41 µg/mL [26]; then, Rot was added to perform the co-treatments.

All the treatments were performed for 24 h, allowing for the reproduction of 40% TH^+^ DOPAn loss in the model, corresponding to the diagnosis in patients, and a significant modulation of all the early markers of TH^+^ DOPAn loss in the model [8]. DMSO was used as the control.

### 4.3. Immunofluorescent Staining of Dopaminergic Neuron

DOPAn demise is responsible for the motor loss in PD. For staining of the DOPAn neurons, we used the well-characterized anti-tyrosine hydroxylase (TH^+^) AB152 antibody (Millipore, Temecula, CA, USA, references available on the supplier website). To further validate the specificity of the signal in our model, we performed a Western blot [73] test, loading a range concentration of whole OBCs-SN lysate (45, 22.5, 11.25, 5.6 μg/lane; 12% acrylamide gel; 1 h 100 V transfer on PVDF membranes; 1 h blocking with 4% milk in 0.2% Tween 20 in 20 mM Tris-HCl pH 7.5; 3 d at 4 °C incubation with the AB152 antibody 1:500; 3× washing; 2 h incubation with the anti-rabbit secondary antibody peroxidase-conjugated (Dako, Agilent Technologies, CA, USA); 3× washing; signal detection by chemiluminescence (ECL-Plus Western blotting Detection Reagents, GE-Healthcare Bio-Science, Italy) on X-ray films (BioMax Light, Kodak Rochester, NY, USA). 

Immunofluorescence was performed as previously described [8]. In brief, at the end of the treatments, slices were fixed in 4% paraformaldehyde for 30 min at RT, washed with PBS, incubated with glycine 0.1 M (Sigma-Aldrich, St. Louis, MO, USA) in PBS for 30 min, rinsed again with PBS, blocked with 10% normal goat serum (NGS, Sigma-Aldrich, St. Louis, MO, USA), 1% bovine serum albumin (BSA, Sigma-Aldrich, St. Louis, MO, USA), and 0.1% Triton-X100 (Sigma-Aldrich, St. Louis, MO, USA) in PBS for 1 h, then incubated with the primary polyclonal antibody AB152 1:250, in incubation solution (1% NGS, 1% BSA, and 0.1% Triton-X100) for 3 d at 4 °C. At the end, slices were rinsed three times in incubation solution and stained with labelled donkey anti-rabbit Alexa fluor 546 secondary anti-body (1:3000, A10040, Life Technologies, Carlsbad, CA, USA) in incubation solution O/N at 4 °C. After washing twice with PBS, slices were stained for 5 min at RT with Hoechst 33,258 (1:10,000, Sigma-Aldrich, St. Louis, MO, USA) in PBS, rinsed with mQ water, and mounted (Fluorescent Mounting Media, Calbiochem, Germany). A DOPAn count, from 3 to 11 biological repetitions, was then performed. Three slices of each condition and biological repetition were used, and all the tyrosine hydroxylase-positive (TH^+^) DOPAn (red signal) in the whole slices were counted, under 20× magnification, by using fluorescent microscopy, Leica DM2000 (Leica Microsystems Srl, Solms, Germany), by 2 researchers who were blinded to the experimental conditions.

### 4.4. RNA Isolation, cDNA Synthesis, and Real-Time PCR Analysis

To assess the effect of UCB on the early molecular mechanism of TH^+^ DOPAn demise in the ex vivo model of PD, we evaluated the previously characterized reported genes [8]. Gene expression from 5 to 10 biological repetitions were analyzed, with the exception of *Cox2* and *Hmox1*, for which 3 to 8 biological repetitions were performed.

Total RNA was extracted using TRI Reagent^®^ (Sigma-Aldrich, St. Louis, MO, USA), according to the manufacturer’s instructions. The concentration and purity of the samples were determined by spectrophotometric validation (230-260-280 nm). Samples passing the purity test were processed for retro-transcription and used in this study. A quantity of 1 μg of total RNA was retro transcribed to complementary DNA (cDNA) with the High-Capacity cDNA Reverse Transcription Kit (Applied Biosystems, Monza, Italy) in a total 20 μL mixture. In each biological repetition, 6 slices were evaluated for each experimental condition. 

Primers [8] were designed using the Beacon Designer 4.2 Software (Premier Biosoft International, Palo Alto, CA, USA) based on a rat sequence available in GenBank. Quantitative real-time PCR (qPCR) was performed in an iQ5 Bio-Rad thermal cycler (BioRad Laboratories, Hercules, CA, USA). Briefly, 25 ng of cDNA and the corresponding gene-specific sense/antisense primers (250 nM each, except Cox2 = 500 nM) were diluted in the Sso Advance SYBER green supermix (Bio-Rad Laboratories, Hercules, CA, USA). The amplification protocol was: 95 °C for 3 min, followed by a 40 times repetition of 95 °C for 20 s, 60 °C for 20 s, and 72 °C for 30 s, followed by the final denaturation at 72 °C for 5 min. The specificity of the amplification was verified by a melting curve analysis, and non-specific products of PCR were not found in any case. The relative quantification was made using iCycleriQ software, version 3.1 (Bio-Rad Laboratories, Hercules, CA, USA) by the ΔΔCt method, taking into account the efficiencies of the individual genes and normalizing the results to the housekeeping genes (TATA-binding protein: *Tbp* and Glyceraldehyde 3-phosphate dehydrogenase: *Gapdh*) [74,75]. 

### 4.5. Glutathione Measurement

To assess the induction of redox imbalance under Rot challenging, and the effects of UCB treatments, reduced (GSH) and oxidized (GSSG) glutathione content were measured in the medium of OBCs-SN, according to the previously described method [76]. The standard curves of GSH (Sigma-Aldrich, St. Louis, MO, USA) and GSSG (Sigma-Aldrich, St. Louis, MO, USA) were used as references. Absorbance was measured using an EnSpire Multimode Plate Reader (PerkinElmer, Waltham, MA, USA). Glutathione level measurements, in 3 to 6 biological repetitions, were performed. The GSH and GSSG levels in the cultures were extrapolated from the readings generated by the standard curves using Curve Expert 1.38 software (Hixon, TN, USA); the GSH/GSSG ratio was then calculated. The GSH and GSSG levels, as well as the GSH/GSSG ratio, were expressed as fold change compared to the control (DMSO treated) slices.

### 4.6. Statistical Analyses

InStat3 for Windows (GraphPad Software, La Jolla, CA, USA) was used to perform statistical analyses. The Shapiro–Wilk normality test was used to study the distribution of the data. Differences among variables, following a parametric distribution, were assessed by a Student *t*-test, whereas those following a non-parametric distribution were assessed with a Mann–Whitney U test. Parametric variables were reported as mean ± standard deviation (S.D.), while non-parametric variables were reported as median, quartile 1,and quartile 3. The correlation between non-parametric variables was investigated using the Spearman Rank Correlation Test, with GraphPad Prism v.5.0.1 (GraphPad Software, La Jolla, CA, USA). For all analyses, the two-sided statistical significance was defined as *p* ˂ 0.05.

## 5. Conclusions

Our work is a proof of concept regarding the protective effect of UCB in PD and most importantly, it provides evidence on the key role of TNF-α in TH^+^ DOPAn demise. This conclusion may be useful for new therapeutic approaches specifically targeting TNF-α to prevent (prophylactic) or to slow (at diagnosis) PD, as well as potentially other neurodegenerative diseases sharing TNF-α as a contributor to neurodegenerative progression. Further experimental work regarding the method of modulating or delivering UCB in the brain will be required to convert these finding for use in clinical settings.

## Figures and Tables

**Table 1:** Spearman’s rank correlation analysis between the TH^+^ DOPAn number and the mRNA level of inflammation, oxidative stress, and neurotrophic growth factor markers. % TH^+^ DOPAn: percentage of TH^+^ dopaminergic neurons count. (A) *Hmox1*: heme-oxygenase 1 and (B) *Srxn1*: sulfiredoxin 1 for redox stress; (C) *Bdnf*: brain-derived neurotrophic factor; (D) *Tnfα*: tumor necrosis factor alpha, (E) *Il6*: interleukin 6; (F) *Cox2*: cyclooxygenase 2 for inflammation.

	*Hmox1*	*Srnx1*	*Bdnf*	*Tnf-α*	*Il6*	*Cox2*
r	−0.8286	−0.7714	−0.6571	−0.7143	−0.6377	−0.2571
*p* Value (two-tailed)	0.0583	0.1028	0.175	0.1361	0.175	0.6583
Significant	no	no	no	no	no	no
